# Comparison of seven cyclosporine A formulations for dry eye disease: A systematic review and network meta-analysis

**DOI:** 10.3389/fphar.2022.882803

**Published:** 2022-11-07

**Authors:** Dongyang Gao, Zhuoli Da, Kan Yang, Yuanyuan Shi

**Affiliations:** ^1^ School of Life Sciences, Beijing University of Chinese Medicine, Beijing, China; ^2^ Department of Ophthalmology, The First People’s Hospital of Lanzhou City, Lanzhou, China; ^3^ Shenzhen Research Institute, Beijing University of Chinese Medicine, Shenzhen, China

**Keywords:** dry eye disease, cyclosporine, commercial formulae, network meta-analysis, meta-analysis

## Abstract

**Background:** Dry eye disease is a common ocular surface disease affecting tens of millions of people worldwide. It is characterized by an unstable tear film and increasing prevalence. Different commercial formulations of cyclosporine A for dry eye have been approved, however, it is still unclear whether the differences in formulations of these products will make a difference in clinical efficacy and safety.

**Methods:** Randomized controlled trials of commercial cyclosporine A formulation for dry eye disease were searched in Pubmed, EMBASE, Scopus, and Cochrane controlled trials registries and Web of Science from inception till 1 December 2021. Independent literature screening, data extraction, quality evaluation, and the study in line with quality standards were analyzed by using Stata16.0 software. The study is registered with PROSPERO under the number CRD42022301423. Code and data for this study is publicly available (https://github.com/DongYangGao/Dongyang.github.io.git).

**Results:** 21 randomized clinical trials with a total of 4,107 participants were included in this study. Restasis^®^ (OR-4.82, 95% CI-6.18 to 3.45, SUCRA 77.2%) was the most effective commercial formulation for reducing OSDI, Zirun^®^ (SUCRA 73.9%) performed better in improving Schirmer’s test. TJ Cyporin^®^ (SUCRA 65.3%) ranked first in terms of improving tear film break-up time. For treatment-emergent adverse events incidence, Clacier^®^ was close to placebo. The risk of reporting bias is considered low.

**Conclusion:** In the comparison of outcomes included in this study, the optimal order of various commercial cyclosporine A formulations is different, so it is difficult to select the optimal formula. Appropriate commercial formulations should be selected according to patients’ conditions in clinical practice.

## Introduction

Dry eye disease (DED), also known as keratoconjunctivitis sicca, is one of the common ocular surface diseases affecting tens of millions of people worldwide (Craig et al., 2017a; Stapleton et al., 2017; Agarwal et al., 2021). Globally, the prevalence of DED in adults is 5%–50% (Stapleton et al., 2017). Changes in the function of the lipid layer on the surface of the eyeball and the quality and/or quantity of tears lead to instability of the tear film, which is an important sign of DED and is often accompanied by ocular irritation, visual impairment, pain or burning (Aragona et al., 2021; Chennakesavalu et al., 2021). Hormonal changes, gender, age, lifestyle, surgical procedures and wearing of contact lenses are related to the onset and deterioration of dry eye (Willcox et al., 2017; Clayton, 2018). DED affects patients’ visual function and quality of life, resulting in increased medical costs and reduced work efficiency, with significant social and economic impacts (Mcdonald et al., 2016; Craig et al., 2017b; Wolffsohn et al., 2017). TFOS DEWS II Pathophysiology Subcommittee proposed that the main mechanism of DED pathophysiology is the vicious inflammatory cycle (Bron et al., 2017). Evaporation and water loss lead to hyperosmolar tissue damage, decreased moisture and humidity on the surface of the eye lead to tear film break up, the instability and hyperosmolar then cause inflammation, malignant inflammatory cycle drives the interaction between the local immune system of the eye and intraocular sensory nerve, causing nerve paresthesia, and the homeostasis of the eye is destroyed and continued circulation (Chen et al., 2010; Belmonte et al., 2017; Yamaguchi, 2018).

**FIGURE 1:**
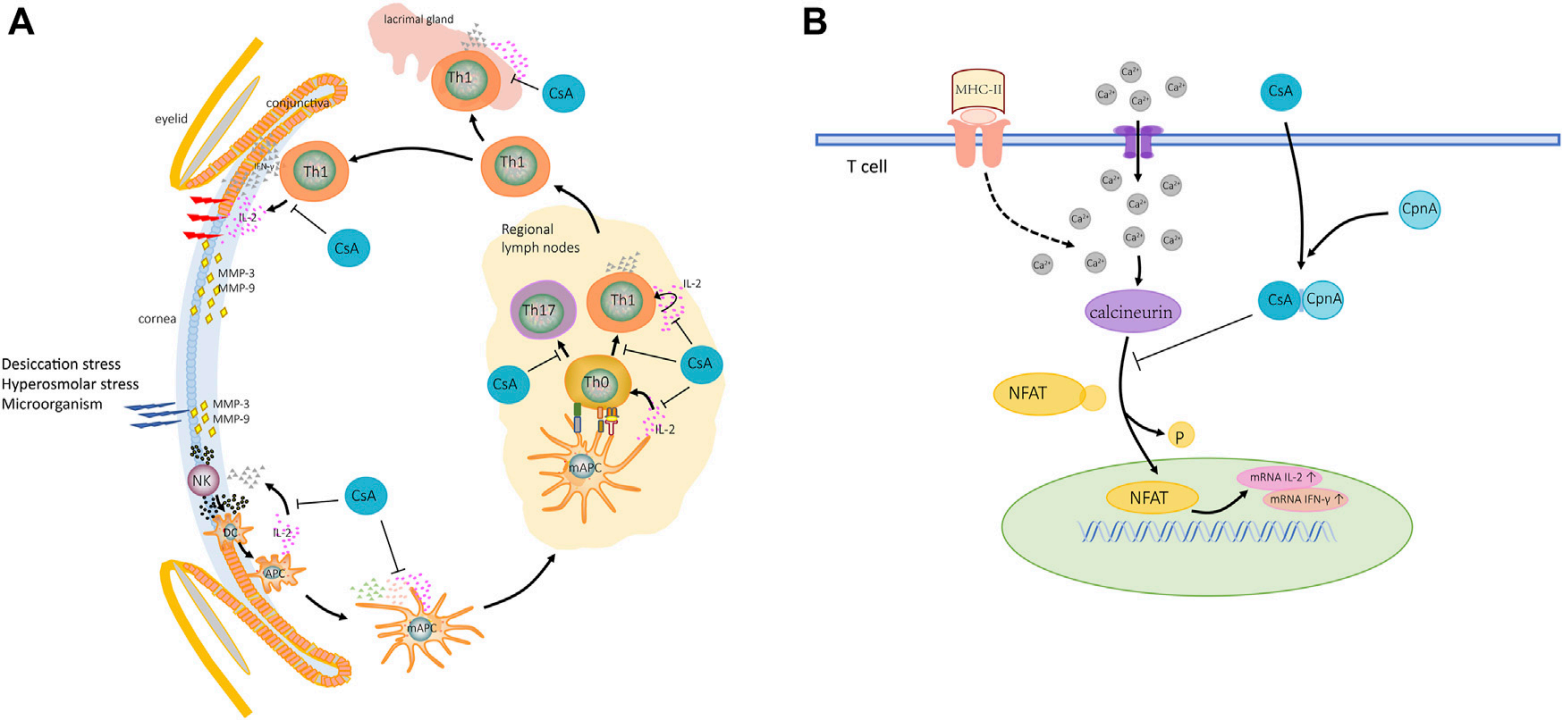
Mechanism of action of CsA for DED. **(A)** The pathogenic factor triggers the ocular surface immune response. In innate immune response, CsA inhibits maturation of DCs and activation and differentiation of T-cell. In adaptive immunity, CsA inhibits secretion of IL-2 from T-cells, reduces the proliferation and infiltration of immune cells in tissues, and the production of MMPs. **(B)** Activated T-cells increased calcium ions in cytoplasm, calcineurin activation, NFAT dephosphorylation, and increased IL-2 and IFN-γ expression. CsA binds CpnA to form complexes, which in combination with calcineurin inhibits the expression of inflammatory factors. Abbreviations: DCs, dendritic cells; NK, natural killer cell; APC, antigen presenting cell; mAPC, mature antigen presenting cell; Th0, naive T-cell; IL-2, interleukin 2; INF-γ, interferon gamma; MMPs, matrix metalloproteinases; TCR, T-cell receptor; NFAT, nuclear factor of activated T-cells; P, phosphorylated; CpnA, cyclophilin A.

Blocking the chronic malignant inflammatory cycle and rebuilding and maintaining the homeostasis of the ocular surface should be the ultimate goal of DED treatment (Baudouin et al., 2016). Topical corticosteroids and cyclosporine should be used for patients with the inefficacy of artificial tears or moderate and severe DED (Beckman et al., 2020; Gupta et al., 2020). Dozens of studies showed that long-term external use of corticosteroids may lead to the risk of ocular hypertension, glaucoma, and cataract (Utine et al., 2010; Agarwal and Rupenthal, 2016; Jones et al., 2017), while preferred immune modulator local cyclosporine A (CsA) could target the chronic inflammatory cycle (Periman et al., 2020) and deal with different underlying pathologic conditions with almost no systemic effect (Pflugfelder, 2004; Baudouin et al., 2016) (Figure 1). CsA is recommended for long-term management of dry eye syndrome.

Restasis^®^ (Allergan, Inc., Irvine, CA, United States), the first commercial topical cyclosporine A ophthalmic emulsion, was approved by the US Food and Drug Administration (FDA) for the treatment of DED in 2002 and has achieved convincing efficacy (Tatlipinar and Akpek, 2005; Bataoel, 2007; Wan et al., 2015). Even so, cyclosporine A is lipophilic and castor oil is used as a solvent, resulting in poor tolerance and low bioavailability (Lallemand et al., 2017; Periman et al., 2020). The need to improve CsA delivery systems has increased in recent years due to the low bioavailability of Restasis^®^, thus, new commercialized registrations apply new technologies and formulations (Periman et al., 2020) such as TJ Cyporin^®^ (which uses nanoemulsion technology to improve bioavailability) (Park et al., 2019; Kang et al., 2020), Ikervis^®^ (which is a cationic nanoemulsion formulation) (Leonardi et al., 2016; Baudouin et al., 2017; Lallemand et al., 2017; Agarwal et al., 2018), Clacier^®^ (which is a transparent nanoemulsion with particle size not exceeding 50 nm) (Kim et al., 2017), Cequa^®^ (which is a transparent aqueous nanomicelle preparation) (Vaishya et al., 2014; Tauber et al., 2018; Goldberg et al., 2019), Zirun^®^ (Chen et al., 2019) and CyclASol^®^ (Gehlsen et al., 2017; Sheppard et al., 2021). The basic information of the seven commercial CsA products is shown in Table 1. Previous studies or reviews (Zhou and WEI, 2014; Wan et al., 2015; Tuan et al., 2020) using paired (head-to-head) comparisons to compare two different formulations of commercial dosage forms, however it is not clear whether the discrepancy in the formulations of these seven products makes a difference in clinical efficacy and safety.

**TABLE 1:** Basic information of seven commercial cyclosporine A products.

Trade name	The company	Approval time	Approval agency	Formula features	Cyclosporine content (%)
Restasis^®^ Tuan et al. (2020)	Allergan Inc., Irvine, CA, United States	2002	FDA	Anionic turbid oil-in-water emulsion	0.05
TJ Cyporin^®^ Park et al. (2019)	Taejoon Pharmaceutical Co., Seoul, Korea	2003	MFDS	Nanoemulsion	0.05
Ikervis^®^ Leonardi et al. (2016)	Santen Pharmaceuticals Co., Ltd., Osaka, Japan	2015	EMA	Cationic emulsion	0.1
Clacier^®^ Kim et al. (2017)	Huons Co., Seongnam, Korea	2016	MFDS	Transparent nanoemulsion with uniform particle size not more than 50 nm	0.05
Cequa^®^ Tauber et al. (2018)	Sun Pharmaceutical Industries, Cranbury, NJ, United States	2018	FDA	Nanomicellar, clear aqueous solution	0.09
Zirun^®^ Chen et al. (2019)	Sinqi Pharmaceutical, Shenyang, China	2020	NMPA	Emulsion	0.05
CyclASol^®^ Zhou and WEI (2014)	Novaliq GmbH, Heidelberg, Germany	2022	NDA	SFA-based nonaqueous preservative-free solution	0.1

FDA, Food and Drug Administration; MFDS, Ministry of Food and Drug Safety; EMA, European Medicines Agency; NMPA, National Medical Products Administration; NDA, New Drug Application; SFA, semifluorinated alkanes.

The aim of the study was to compare and rank the effectiveness and safety of different cyclosporine A formulations for the treatment of dry eye using existing datasets (Rochwerg et al., 2018). We then designed and conducted a network meta-analysis (Huang et al., 2016), which combines direct and indirect evidence to compare multiple interventions at the same time in the presence of high-quality placebo-controlled trials (Gao et al., 2021) to increase the accuracy of results to guide clinical practice (Cipriani et al., 2018).

## Methods

### Search strategy

Pubmed, EMBASE, Scopus, and Cochrane Controlled Trials Registries and Web of Science for all potential RCTs were searched. Additionally, ClinicalTrials.gov was searched for unpublished trials. The search period is from inception of these libraries up till 1 December 2021 with no restrictions on source or language. Keywords (MeSH in PubMed and Emtree in Embase) and free words are used for retrieval: 1) Dry Eye Syndrome, Dry Eye Disease, Dry Eye, Evaporative Dry Eye Disease, Evaporative Dry Eye Syndrome; 2) Cyclosporine, Cyclosporine A, Cyclosporin A, Ciclosporin, Restasis, Ikervis, Clacier, Cequa, OTX-101, Zirun, TJ Cyporin, Cyporin N, Cyclosporine Nanoemulsion, CyclASol, Cyclosporine A cationic emulsion, 0.1% Cyclosporine, 0.05% Cyclosporine, 0.09% Cyclosporine; 3) Randomized controlled trial, randomized, placebo. Heading terms AND free words in each group are linked by “OR”, AND three groups are combined by “AND”. The complete search strings for all databases retrieved are provided in Supplementary Table S2.

### Inclusion and exclusion criteria

According to our objective, retrieved articles that meet the following criteria will be included in the meta-analysis: 1) Study design: all randomized controlled studies (RCTs) that compare commercial CsA with placebo or vehicle for the treatment of dry eye, and have access to complete data. 2) Participants: All patients clinically diagnosed with DED were not limited by age, region, gender, race, or other factors. 3) Type of intervention: The intervention in the experimental group was topical with different types of commercial CsA with or without artificial tears and placebo. 4) Type of comparison: The control group could be treated with artificial tears, excipients, or placebo in addition to CsA. 5) Outcome of dry eye intervention, such as OSDI score, Schirmer’s test (ST) with or without anesthesia on, tear film break-up time (BUT), and Treatment-emergent adverse events (TEAEs).

Studies were excluded if they met one of the following criteria: 1) observational studies, non-randomized controlled trials, and real-world studies. 2) All animal studies and cadaver studies. 3) All reviews, letters, case reports, conference summaries or records, systematic reviews, and meta-analyses. 4) Low-quality studies were assessed according to the Cochrane Manual. 5) The outcome data could not be extracted, nor could they be calculated according to the graphs in the article, or the studies obtained by contacting the authors.

### Data extraction

Two reviewers (GDY and DZL) extracted independently from the full text of the studies that met the screening criteria. After re-checking with Endnote X9 for Windows (Thomson Reuters, United States) literature management software, the preliminary screening was completed by reading the titles and abstracts, and the full text of potential studies was read to determine whether to include them. If necessary, the authors of the original study can be contacted by email or phone to obtain information of critical importance. All information was independently extracted into a Microsoft Excel spreadsheet, including, if any, country of origin, first author, year of publication, study type, a sample size of patients included, diagnostic criteria, interventions, outcome measures, and baseline information and outcome data were extracted into a standardized form. Results are checked back-to-back and any discrepancies can be resolved by referring to the original study or consulting a third reviewer (SYY).

### Risk of bias assessment

Two reviewers (GDY and DZL) performed independent quality evaluations of the included studies, and the Cochrane Collaboration Risk of Bias tool (Higgins et al., 2011) was used to assess: Random sequence generation, allocation hiding, blinding of participants and personnel, blinding of outcome evaluation, incomplete outcome data, selective reporting, and other biases. Each study is assessed as low risk, high risk, or unclear risk. Any differences are resolved through discussion or consultation with a third independent examiner (SYY).

### Statistical analysis

Our network meta-analysis was designed and conducted by NMA’s Systematic evaluation and The Preferred Report Project (PRISMA) Reporting Guidelines for Meta-Analysis (Hutton et al., 2015) (Supplementary Table S1). Our team registered the master agreement on PROSPERO, with the registration number CRD42022301423. The method described in this study was accomplished using Stata 16.0 Software, and the data and code for the analysis can be accessed from our Giuhub Repositories (https://github.com/DongYangGao/Dongyang.github.io.git).

Odds ratio (OR) was used as effect size and 95% confidence interval (CI) was calculated. Stata 16.0 software network group command data preprocessing. The inconsistency test is mainly used to evaluate the difference between direct and indirect comparison results. When there is a closed ring, the consistency test is carried out by the node analysis method, if *p* > 0.05, indicating good consistency, the consistency model was used for analysis; otherwise, the inconsistency model was used for analysis. A network diagram of different outcome indicators was drawn for comparison between different cyclosporine A products. Dot area represented the number of clinical trial participants using the product, and the thickness of the line between dots represented the number of included studies (Salanti et al., 2011). The surface under cumulative ranking (SUCRA) represents the overall probability that an intervention is one of the best treatments based on the ranking of all interventions. SUCRA is expressed as a percentage. When SUCRA is 100%, intervention is effective; when SUCRA is 0, intervention is ineffective (Cope and JANSEN, 2013; Shim et al., 2017). Finally, a funnel plot is used to identify the existence of a small sample effect.

## Results

### Literature retrieval and inclusion features

**FIGURE 2:**
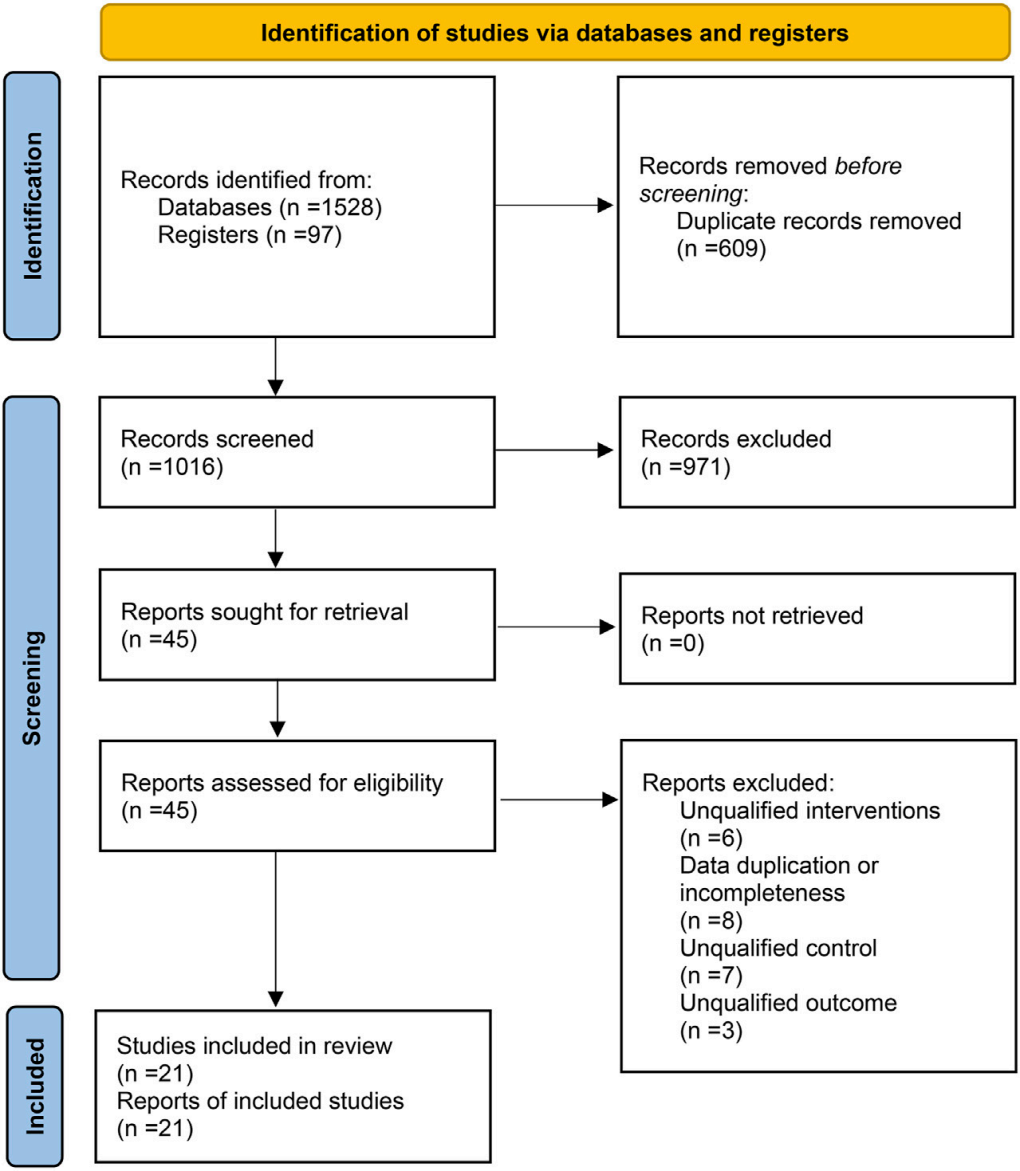
Literature retrieval process.

A total of 1,528 articles were retrieved from the electronic database, 512 duplicate studies were deleted, and 971 articles were excluded after reading titles and abstracts. After reading the full text of the 45 articles, 24 of the studies were excluded according to exclusion criteria, such as seven studies that did not meet the criteria that “controls should be treated with artificial tears, excipients, or placebo.” Finally, 21 eligible studies were included. The literature retrieval process (Page et al., 2021) is shown in Figure 2.

The 21 studies that were eventually included were published between 2000 and 2021 and were shown to have been conducted globally, with seven in Europe (including two in collaboration between the United States and Germany), eight in Asia, and six in the United States alone. A total of 4,107 participants were recruited and followed for 8 weeks to 6 months. All studies included adults older than 18 years of age. All studies included at least one outcome measure for comparison. Table 2 described characteristics of these included studies.

**TABLE 2:** Basic features of the included studies.

Study	Year	Country	Interventions and control		Number of patients (baseline)	Mean age (SD)	Duration	Outcomes reported
Sall et al. (2020)	2000	United States	Restasis^®^	BID	293	58.7 (13.9)	6 months	③④
			Artificial tears	BID	292	59.9 (14.3)		
Stevenson et al. (2000)	2000	United States	Restasis^®^	BID	33	N/A	12 weeks	④
			Artificial tears	BID	31	N/A		
Perry et al. (2006)	2006	United States	Restasis^®^	BID	16	N/A	3 months	②③④
			Artificial tears	BID	17	N/A		
Willen et al. (2008)	2008	United States	Restasis^®^	BID	22	44.0 (12.6)	3 months	①②③
			Artificial tears	BID	22	42.2 (14.8)		
Kim et al. (2009)	2009	Korea	Restasis^®^	BID	50	41.3 (9.7)	3 months	②③④
			Artificial tears	QID	50	35.9 (8.5)		
Guzey et al. (2009)	2009	Turkey	Restasis^®^	BID	32	61.5 (6.9)	6 months	①②③
			Vehicle	BID	32	60.5 (8.2)		
Altiparmak et al. (2010)	2010	Turkey	Restasis^®^	BID	25	41.0 (1.1)	6 months	②③④
			Artificial tears	BID	48	40.9 (8.8)		
Chen et al. (2010)	2010	China	Restasis^®^	BID	116	46.6 (11.1)	8 weeks	②③④
			Vehicle	BID	117	46.0 (12.1)		
Rao (2010)	2010	China	Restasis^®^	BID	41	47.5 (5.9)	12 months	①②③④
			Artificial tears	BID	33	48.2 (6.3)		
Demiryay et al. (2011)	2011	Turkey	Restasis^®^ +Artificial Tears	BID	22	46.6 (12.3)	4 months	②③④
			Artificial Tears	QID	20	44.3 (14.4)		
Prabhasawat et al. (2012)	2012	Thailand	Restasis^®^	BID	36	48.1 (13.9)	12 weeks	①②④
			Artificial tears	BID	34	55.0 (13.0)		
Kang et al. (2020)	2019	Korea	TJ Cyporin^®^	BID	18	55.1 (13.5)	12 weeks	①②③④
			Restasis^®^	BID	18	53.5 (9.7)		
Park et al. (2019)	2019	Korea	TJ Cyporin^®^	BID	58	N/A	12 weeks	①②③④
			Restasis^®^	BID	58	N/A		
Leonardi et al. (2016)	2016	9 European countries	Ikervis^®^	QD	154	60.8 (13.5)	6 months	①②③④
			Vehicle	QD	91	62.1 (11.8)		
Baudouin et al. (2017)	2017	6 European countries	Ikervis^®^	QD	241	57.6 (12.9)	6 months	②④
			Vehicle	QD	248	58.8 (12.7)		
Kim et al. (2017)	2017	Korea	Clacier^®^	BID	34	N/A	12 weeks	①②③④
			Restasis^®^	BID	39	N/A		
Tauber et al. (2018)	2018	United States	Cequa^®^	BID	152	59.2 (14.6)	12 weeks	④
			Vehicle	BID	152	59.3 (13.8)		
Goldberg et al. (2019)	2019	United States	Cequa^®^	BID	371	58.4 (14.1)	12 weeks	④
			Vehicle	BID	373	59.5 (14.7)		
Chen et al. (2019)	2019	China	Zirun^®^	BID	119	46.3 (12.5)	12 weeks	①②③④
			Vehicle	BID	115	45.0 (12.4)		
Wirta et al. (2019)	2019	The United States and Germany	CyclASol^®^	BID	51	64.3 (10.7)	16 weeks	①④
			Restasis^®^	BID	53	62.8 (11.9)		
			Vehicle	BID	52	61.3 (10.5)		
Sheppard et al. (2021)	2021	The United States and Germany	CyclASol^®^	BID	162	61.5 (13.6)	12 weeks	③④
			Vehicle	BID	166	61.3 (12.7)		

Vehicle (the same ophthalmic emulsion formulation without cyclosporine); N/A, data not available; ① Ocular surface disease index (OSDI) score; ② Schirmer’s test (ST) with or without anesthesia; ③ Tear film break-up time (BUT); ④ Treatment-Emergent AEs (TEAEs).

### Risk assessment of bias

**FIGURE 3:**
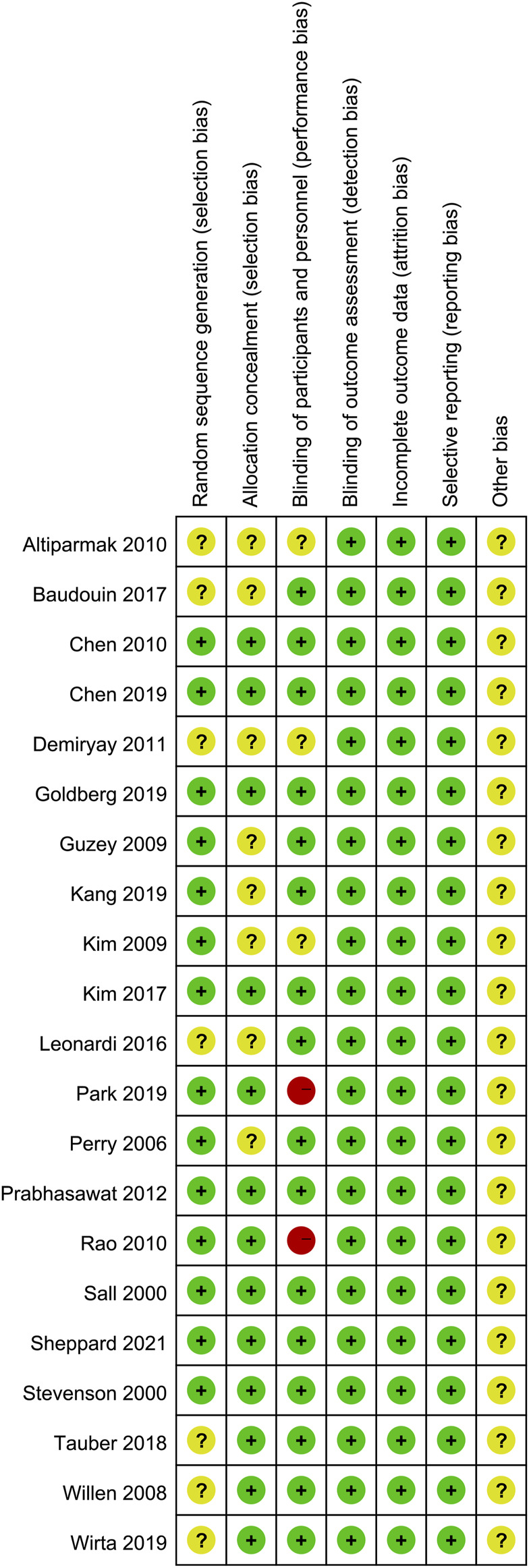
Literature bias risk assessment results.

The risk of bias was assessed for 21 included studies (Figure 3). For selection bias, all included studies were randomized, but seven studies (Willen et al., 2008; Altiparmak et al., 2010; Demiryay et al., 2011; Leonardi et al., 2016; Baudouin et al., 2017; Tauber et al., 2018; Wirta et al., 2019) did not describe the specific generation method of random sequences. Eight studies (Perry et al., 2006; Guzey et al., 2009; Kim et al., 2009; Altiparmak et al., 2010; Demiryay et al., 2011; Leonardi et al., 2016; Baudouin et al., 2017; Kang et al., 2020) did not provide detailed information about allocation hiding methods, and were all unable to determine the choice bias and rated as “unclear risk.” In terms of implementation bias and detection bias, three studies (Kim et al., 2009; Altiparmak et al., 2010; Demiryay et al., 2011) did not report the use of the blind method and were rated as “unclear risk,” and two studies (Rao, 2010; Park et al., 2019) were rated as “high risk” because researchers were single-blind. All 21 studies were considered to have a low risk of loss of follow-up bias because the number of participants who dropped out of the study was reported, and all studies reported all outcome measures described in their respective methods, with no bias reported. None of the 21 studies described other bias in detail and was rated as “unclear risk.”

### Ocular surface disease index score change from baseline

**FIGURE 4:**
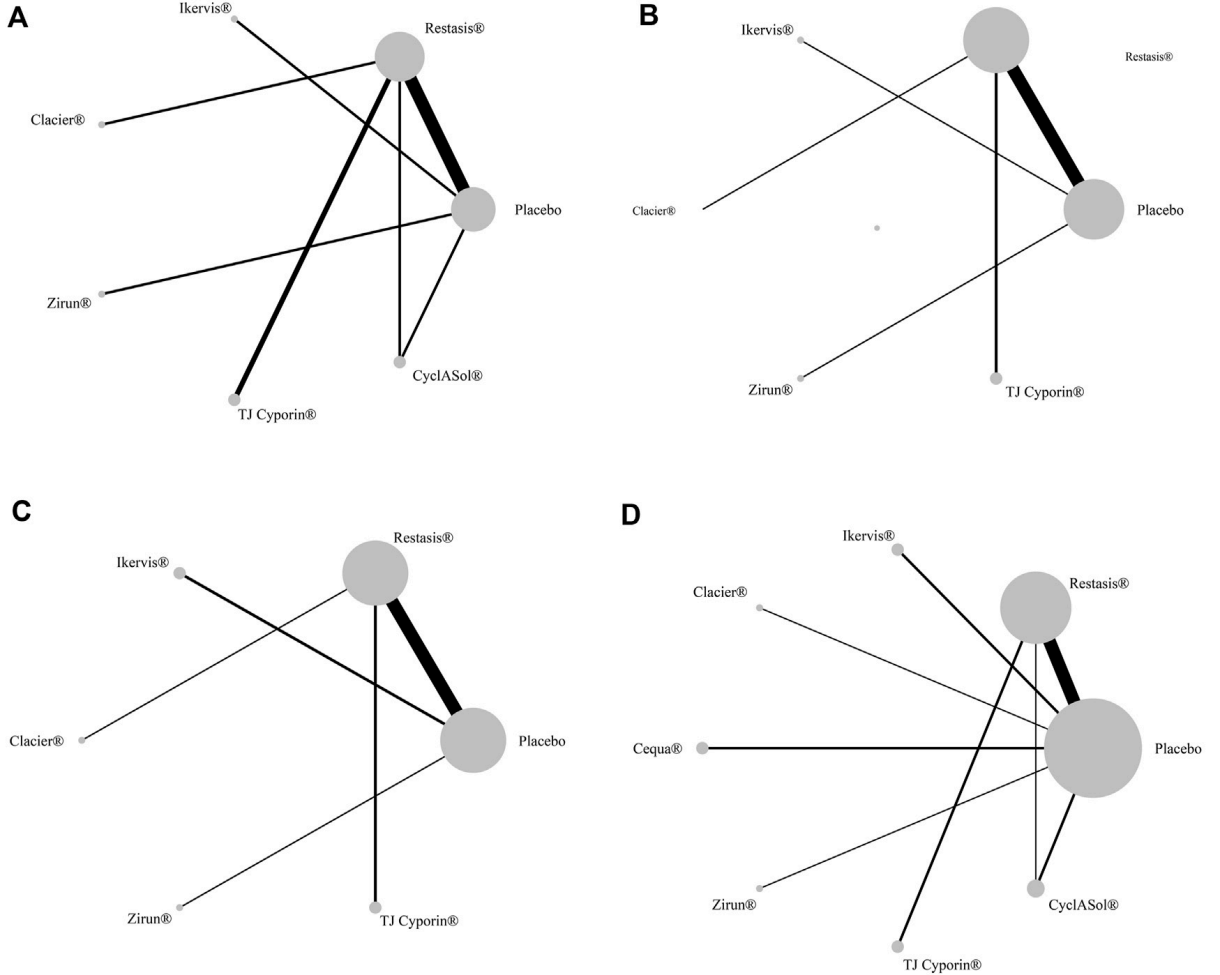
Network comparison of the four indicators. **(A)**: ocular surface disease index (OSDI) change from baseline; **(B)**: Schirmer’s test (ST) change from baseline; **(C)**: tear film break-up time (BUT) change from baseline; **(D)**: Treatment-emergent AEs (TEAEs). The node size represents the sample size of intervention measures, and the line represents the number of RCTs between the two intervention measures.

**FIGURE 5:**
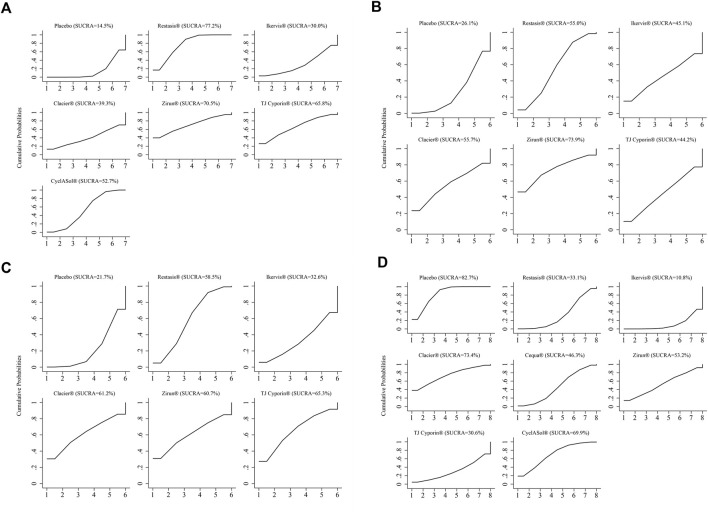
Surface plot under the cumulative ranking curve (SUCRA) of all outcome measures. **(A)**: ocular surface disease index (OSDI) change from baseline; **(B)**: Schirmer’s teat (ST) change from baseline; **(C)**: tear film break-up time (BUT) change from baseline; **(D)**: Treatment-emergent AEs (TEAEs). A larger SUCRA score indicates that the intervention is more effective.

**FIGURE 6:**
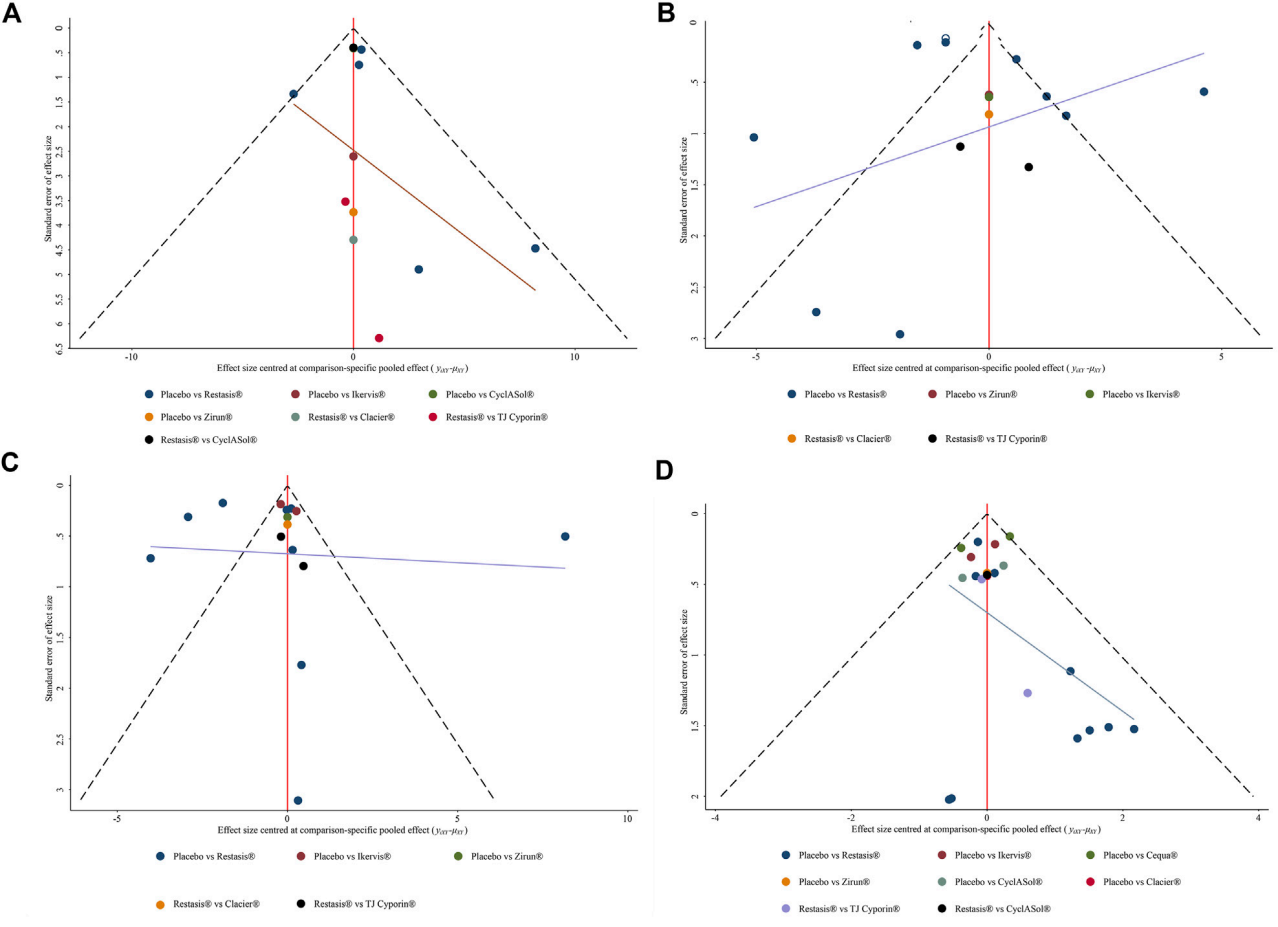
Comparison-adjusted funnel plot for all outcome measures. The red line represents the invalid hypothesis. Different colors correspond to different comparisons. **(A)**: ocular surface disease index (OSDI) change from baseline; **(B)**: Schirmer’s test (ST) change from baseline; **(C)**: tear film break-up time (BUT) change from baseline; **(D)**: Treatment-emergent AEs (TEAEs).

Ten studies with a total of 1,090 participants reported changes in OSDI scores from baseline across eight treatments, as shown in Figure 4. The changes of Restasis^®^ (OR-4.82, 95% CI-6.18 to −3.45) and CyclASol^®^ (OR-3.40, 95% CI-4.94 to −1.86) from baseline were significantly lower than those of Placebo. Other comparisons found no significant difference. A league chart showing the relative impact of different formulations is shown in Table 3. The SUCRA probability ranking of all treatments with reduced OSDI score showed that Restasis^®^ may be the most effective commercially available formulation. The ranking result of SUCRA probability from high to low is Restasis^®^ > Zirun^®^ > TJ Cyporin^®^ > CyclASol^®^ > Clacier^®^ > Ikervis^®^ > Placebo. The details are shown in Figure 5. The comparison adjustment funnel of OSDI score changes is shown in Figure 6, and no significant visual asymmetry is found.

**TABLE 3:** League table of results for OSDI and ST score change from baseline.

ST score change from baseline							
**OSDI score change from baseline**	**Restasis** ^®^	1.73 (−3.81, 7.27)	0.45 (−3.53, 4.42)	N/A	0.36 (−4.96, 5.68)	0.48 (−5.07, 6.03)	1.18 (−0.68, 3.04)
	0.51 (−7.07, 8.09)	**Zirun** ^®^	2.18 (−4.64, 9.00)	N/A	1.37 (−6.31, 9.05)	2.21 (−5.18, 9.60)	2.91 (−2.31, 8.13)
	−0.32 (−6.45, 5.81)	−0.83 (−10.57, 8.91)	**TJ Cyporin** ^®^	N/A	0.81 (−5.83, 7.45)	−0.03 (−6.86, 6.79)	0.73 (−3.65, 5.12)
	−1.42 (−2.96, 0.12)	−1.93 (−9.55, 5.68)	−1.10 (−7.41, 5.21)	**CyclASol®**	N/A	N/A	N/A
	−3.14 (−11.68, 5.40)	−3.65 (−15.07, 7.76)	−2.82 (−13.33, 7.69)	−1.72 (−10.39, 6.96)	**Clacier®**	0.84 (−6.85, 8.53)	1.54 (−4.10, 7.18)
	−3.72 (−9.18, 1.75)	−4.23 (−13.37, 4.91)	−3.40 (−11.60, 4.80)	−2.30 (−7.81, 3.21)	−0.58 (−10.71, 9.56)	**Ikervis®**	0.70 (−4.53, 5.93)
	−4.82 (−6.18,−3.45)	−5.33 (−12.79, 2.13)	−4.50 (−10.76, 1.77)	**−3.40 (−4.94, −1.86)**	−1.68 (−10.32, 6.97)	−1.10 (−6.39, 4.19)	**Placebo**

Each cell contains the odds ratio (OR) and 95% confidence interval for OSDI changes and ST changes; comparisons should be read from left to right. Bold numbers indicate statistically significant differences. 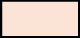
OSDI score change from baseline, 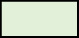
ST score change from baseline; N/A, data not available.

### Schirmer’s test score changes

Fourteen studies with a total of 1,913 participants reported changes in ST scores involving seven treatments, as shown in Figure 4. There was no significant difference in baseline changes in ST scores between treatments. A league chart showing the relative effects of different treatments is shown in Table 3. The SUCRA probability ranking results for all treatments that improved ST scores showed that Zirun^®^ was probably the most effective commercially available formulation, with the SUCRA probability ranking from high to low as Zirun^®^ > Clacier^®^ > Restasis^®^ > Ikervis^®^ > TJ Cyporin^®^ > Placebo; The details are shown in Figure 5. The comparison adjustment funnel plot of ST score changes is shown in Figure 6. The funnel plot results show poor symmetry, suggesting that there may be a certain publication bias.

### Tear film break-up time changes from baseline

Fifteen studies with a total of 1,881 participants reported the results of changes in BUT involving seven treatments, as shown in Figure 4. There was no significant difference in BUT among all comparisons. A league chart showing the relative impact of different formulations is shown in Table 4. The SUCRA probability ranking of all formulations that improved BUT scores showed that TJ Cyporin^®^ was probably the most efficient commercial formulation, and the SUCRA probability ranking from high to low was TJ Cyporin^®^ > Clacier^®^ > Zirun^®^ > Restasis^®^ > Ikervis^®^ > Placebo. The details are shown in Figure 5. The comparison adjustment funnel diagram of OSDI score changes is shown in Figure 6. The funnel diagram results show poor symmetry, suggesting that there may be a certain publication bias.

**TABLE 4:** League table of results for BUT score change from baseline and TEAEs.

BUT change from baseline								
**Treatment-emergent AEs**	**Placebo**	−0.02 (−1.04, 1.01)	−0.13 (−0.78, 0.52)	−0.35 (−1.34, 0.64)	−0.44 (−0.92, 0.05)	−0.62 (−1.10,−0.14)	−0.74 (−1.84, 0.36)	−0.99 (−1.52,−0.46)
	2.41 (−3.78, 8.60)	**Clacier®**	−0.12 (−1.33, 1.10)	−0.33 (−1.76, 1.09)	**−0.42 (−1.55, 0.71)**	**−0.60 (−1.74, 0.53)**	**−0.72 (−2.22, 0.78)**	**−0.98 (−2.13, 0.18)**
	N/A	N/A	**CyclASol®**	**−0.22 (−1.40, 0.97)**	**−0.30 (−1.12, 0.51)**	**−0.49 (−1.22, 0.25)**	**−0.60 (−1.84, 0.63)**	**−0.86 (−1.70, −0.02)**
	2.55 (−4.02, 9.12)	0.14 (−8.89, 9.16)	N/A	**Zirun®**	−0.09 (−1.19, 1.01)	**−0.27 (−1.37, 0.83)**	−0.39 (−1.87, 1.09)	**−0.64 (−1.77, 0.48)**
	N/A	N/A	N/A	N/A	**Cequa®**	**−0.18 (−0.88, 0.52)**	**−0.30 (−1.51, 0.92)**	**−0.55 (−1.27, 0.16)**
	1.90 (−0.26, 4.06)	0.51 (−6.04, 7.07)	N/A	0.65 (−5.55, 6.85)	N/A	**Restasis®**	**−0.12 (−1.10, 0.86)**	**−0.37 (−1.10, 0.35)**
	2.62 (−2.33, 7.57)	0.21 (−7.71, 8.13)	N/A	0.07 (−7.56, 7.71)	N/A	0.72 (−3.73, 5.17)	**TJ Cyporin®**	**−0.26 (−1.48, 0.97)**
	0.27 (−4.10, 4.63)	2.14 (−5.43, 9.71)	N/A	2.28 (−5.61, 10.16)	N/A	1.63 (−3.24, 6.50)	2.35 (−4.25, 8.95)	**Ikervis®**

Each cell contains the odds ratio (OR) and 95% confidence interval for BUT changes and TEAEs; the comparison should be read from left to right. Bold numbers indicate statistically significant differences. 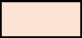
BUT score change from baseline, 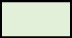
ST score change from baseline; N/A, data not available.

### Treatment-emergent AEs

Nineteen studies with a total of 4,032 participants reported the results of TEAEs, involving eight treatments, as shown in Figure 4. The league chart of the relative effects of the treatments is shown in Table 4. Placebo (SUCRA, 82.7%) showed the lowest incidence of TEAEs compared to the other formulations except for Clacier^®^, and the difference was significant. There was no significant difference between Placebo (OR-0.02, 95% CI-1.04 to 1.01) and Clacier^®^. The SUCRA probability of TEAEs incidence in each treatment ranked from high to low as Placebo > Clacier^®^ > CyclASol^®^ > Zirun^®^ > Cequa^®^ > Restasis^®^ > TJ Cyporin^®^ > Ikervis^®^ (Figure 5). The comparison adjustment funnel diagram of TEAEs is shown in Figure 6, and no significant visual asymmetry is found.

## Discussion

To our knowledge, this is the first study to comprehensively compare the efficacy and safety of different commercial cyclosporine A formulations in the treatment of the dry eye. Previous systematic evaluations have shown that although local CsA can improve some objective and subjective outcomes of patients with dry eye, there will be an inconsistent improvement of outcome indicators and an increase in treatment-emergent AEs (Zhou and WEI, 2014; Wan et al., 2015; De Paiva et al., 2019; Tuan et al., 2020). To weigh the pros and cons of different types of commercial CsA and help clinicians make decisions, we compared different application strategies of direct or indirect evidence, using frequency theory framework network meta-analysis, screening of RCT, participants included 21 eligible studies, evaluated the four outcome indicators: OSDI score changes, ST score changes, (BUT) changes, treatment-emergent AEs (TEAEs) incidence. The ranking of all formulations and the accuracy of estimation was obtained (Dias et al., 2013).

Topical use of cyclosporine A is a highly effective treatment strategy for direct exposure to the surface of the eye. However, due to the low bioavailability of the eye for the sake of its good protective mechanisms (eye barrier, tear dilution, blinking and tear removal) (Davies, 2000; Gaudana et al., 2010), and the high lipophilic nature of CsA, the toxicity shown by the use of osmotic enhancers and surfactants in formulations and the discomfort caused by oil-based formulations (Cholkar et al., 2012; Rodriguez-Aller et al., 2013), formula reform is imperative. Currently, these products are only approved for marketing in some regions (Lallemand et al., 2017), and it is not clear whether the differences in formulations translate into differences in clinical efficacy and safety (Tong et al., 2020).

Our network meta-analysis of 4,107 participants showed that Restasis^®^, Zirun^®^, TJ Cyporin^®^, CyclASol^®^, Clacier^®^, and Ikervis^®^ were more effective than placebo on three subjective and objective measures of effectiveness: OSDI score, ST, and BUT. Although Cequa^®^ has completed phase 2/3 and Phase 3 trials, it could not be included because the outcome measure was the number of people who improved. Restasis^®^ (OR-4.82, 95% CI-6.18 to 3.45, SUCRA 77.2%) was the most effective formulation for reducing OSDI, superior to other commercially available formulations, and the difference was significant. OSDI questionnaire evaluates subjective symptoms in patients with dry eye (Grubbs et al., 2014; Pult and WOLFFSOHN, 2019). Dryness and discomfort were the symptoms that scored highest on the questionnaire (Begley et al., 2002). Restasis^®^ (Allergan Inc., Irvine, CA), the first commercial CsA emulsion, was used for the treatment of DED (Tatlipinar and Akpek, 2005). It is a preservative-free anionic oil-in-water nanoemulsion with castor oil as solvent, polysorbate 80 as an emulsifier, and carbomer copolymer as a stabilizer (Lallemand et al., 2017). The advantage of Restasis^®^ in improving subjective symptoms is mainly due to the maturity of its preparation process, which is consistent with previous literature reports (Tong et al., 2020).

The application of new excipients (such as semi-fluorinated Alkanes) and the change of dosage form (like cationic emulsion and nano-micellar aqueous solution) are the main directions. Zirun^®^(Sinqi Pharmaceutical, Shenyang, China) uses new micelles as nanocarriers for drug delivery (Yu et al., 2018) and is an ophthalmic emulsion approved by NMPA in China in 2020 (Chen et al., 2019). Zirun^®^ (SUCRA 73.9%) was the best choice for improving Schirmer’s Test (ST). ST primarily assesses the secretion of basic tear and the function of the main lacrimal gland developed in 1903 (Erickson et al., 1958; Li et al., 2012). According to current information disclosed by Zirun^®^, the retention effect of the new micellar preparation in the eye is 4.5 times higher than that of the traditional cyclosporine A preparation (Yu et al., 2018), which may play A major role in repairing lacrimal gland function. Our study also shows that TJ Cyporin^®^ (SUCRA 65.3%) ranked first in terms of improved BUT values. The dropper size is 20 nm–200 nm, with acceptable stability and bioavailability (Lallemand et al., 2017). Tear film instability may be the relative abnormality of the mucin/water layer attached to calyx glycose (Tsubota, 2018). Similar to previous reports, TJ Cyporin^®^ has an obvious repair function on calyx glycose in previous reports (Kang et al., 2020), so it makes sense. For security indicator TEAEs, placebo was unquestionably the lowest. Our results also showed no difference in safety between Clacier^®^ (SUCRA 73.4%) and placebo. All dosage forms have been reported to cause certain adverse reactions (Leonardi et al., 2015), but study with similar results have been analyzed that hydrophilic agent (ethylene oxide) used in Clacier^®^ forms nano-emulsion with small and uniform particle sizes, may reduce irritation and blur (Kang et al., 2020).

There are some limitations to this study. First, two of the included studies were single-blind and rated as high risk, which may have a certain bias. Second, although authoritative databases and registered websites were selected, RCTs for which we did not find commercial CsA formulations for the dry eye could not be included due to language or literature publication restrictions in some countries. Third, there are some confounding factors in the outcomes that may affect the stability of the results. For example, OSDI evaluation is subjective to a certain extent, and ST and BUT test personnel may have certain experience and technical deviations. Fourth, some dosage forms are once a day, while others are twice a day. This difference in the frequency of dosage use may cause some uncertainty, and future studies with larger sample sizes will be required to conduct further analysis of the difference in the frequency of dosage. Due to some differences in the baseline characteristics of the included trials, the selection of formulations determined by disease characteristics cannot be fully confirmed. In future studies, subgroup analyses based on different baseline characteristics should be feasible after the inclusion of high-quality randomized controlled studies. In addition, we have not found any cost-benefit comparison between different formulations at present, and the advantages and disadvantages of different CsA formulations should be further explored and compared from the perspective of health economics in the future. Finally, if other immunosuppressants can be included in a larger range of statistical comparison, more statistical results may be obtained.

In summary, the network meta-analysis of this study was designed to resolves discrepancies between published studies, the results of this network meta-analysis suggest that various commercial formulations of CsA have good efficacy in the treatment of patients with dry eye. Restasis^®^ is the best choice for reducing the Ocular Surface Disease Index (OSDI) score. Zirun^®^ and TJ Cyporin^®^ were the most effective in improving Schirmer’s Test (ST) and tear film break-up time (BUT) values, respectively. In terms of safety, Clacier^®^ is similar to placebo, although other dosage forms may be associated with some adverse effects. The optimal order of various commercial CsA formulations was different among individual outcomes, so it was difficult to select the optimal formula. More double-blind, multi-center, large-sample, and high-quality clinical trials are still needed for supplementary validation to provide stronger evidence support.

## Data Availability

The original contributions presented in the study are included in the article/Supplementary Material, further inquiries can be directed to the corresponding authors.
