# Supplementary material for: Comparison of seven cyclosporine A formulations for dry eye disease: A systematic review and network meta-analysis
Source: Front Pharmacol. 2022 Nov 7;13:882803. doi: 10.3389/fphar.2022.882803 (PMC9676501; doi:10.3389/fphar.2022.882803)
Supplement: Supplementary file 1 [file Table1.DOCX]

Table 1 Basic information of seven commercial cyclosporine A products

| Trade name | The company | Approval time | Approval agency | Formula features | Cyclosporine content |
| --- | --- | --- | --- | --- | --- |
| Restasis®^[40]^ | Allergan Inc, Irvine, CA | 2002 | FDA | Anionic turbid oil-in-water emulsion | 0.05% |
| TJ Cyporin®^[28]^ | Taejoon Pharmaceutical Co., Seoul, Korea | 2003 | MFDS | Nanoemulsion | 0.05％ |
| Ikervis®^[31]^ | Santen Pharmaceuticals Co., Ltd., Osaka, Japan | 2015 | EMA | Cationic emulsion | 0.1% |
| Clacier®^[33]^ | Huons Co., Seongnam, Korea | 2016 | MFDS | Transparent nanoemulsion with uniform particle size not more than 50nm | 0.05% |
| Cequa®^[35]^ | Sun Pharmaceutical Industries, Cranbury, NJ, USA | 2018 | FDA | Nanomicellar, clear aqueous solution | 0.09% |
| Zirun®^[37]^ | Sinqi Pharmaceutical, Shenyang, China | 2020 | NMPA | Emulsion | 0.05% |
| CyclASol®^[41]^ | Novaliq GmbH, Heidelberg, Germany | 2022 | NDA | SFA-based nonaqueous preservative-free solution | 0.1% |

FDA, Food and Drug Administration; MFDS, Ministry of Food and Drug Safety; EMA, European Medicines Agency; NMPA, National Medical Products Administration; NDA, New Drug Application; SFA, semifluorinated alkanes.

Table 2 Basic features of the included studies

| Study | Year | Country | Interventions and control | | Number of patients (baseline) | Mean age (SD) | Duration | Outcomes reported |
| --- | --- | --- | --- | --- | --- | --- | --- | --- |
| Sall *et al.*^[47]^ | 2000 | United States | Restasis® | BID | 293 | 58.7 (13.9) | 6 months | ③④ |
|  |  |  | Artificial tears | BID | 292 | 59.9 (14.3) |  |  |
| Stevenson *et al.*^[48]^ | 2000 | United States | Restasis® | BID | 33 | N/A | 12 weeks | ④ |
|  |  |  | Artificial tears | BID | 31 | N/A |  |  |
| Perry *et al.*^[49]^ | 2006 | United States | Restasis® | BID | 16 | N/A | 3 months | ②③④ |
|  |  |  | Artificial tears | BID | 17 | N/A |  |  |
| Willen *et al.*^[50]^ | 2008 | United States | Restasis® | BID | 22 | 44.0 (12.6) | 3 months | ①②③ |
|  |  |  | Artificial tears | BID | 22 | 42.2 (14.8) |  |  |
| Kim *et al.*^[51]^ | 2009 | Korea | Restasis® | BID | 50 | 41.3 (9.7) | 3 months | ②③④ |
|  |  |  | Artificial tears | QID | 50 | 35.9 (8.5) |  |  |
| Guzey *et al.*^[52]^ | 2009 | Turkey | Restasis® | BID | 32 | 61.5 (6.9) | 6 months | ①②③ |
|  |  |  | Vehicle | BID | 32 | 60.5 (8.2) |  |  |
| Altiparmak *et al.*^[53]^ | 2010 | Turkey | Restasis® | BID | 25 | 41.0 (1.1) | 6 months | ②③④ |
|  |  |  | Artificial tears | BID | 48 | 40.9 (8.8) |  |  |
| Chen *et al.*^[14]^ | 2010 | China | Restasis® | BID | 116 | 46.6 (11.1) | 8 weeks | ②③④ |
|  |  |  | Vehicle | BID | 117 | 46.0 (12.1) |  |  |
| Rao *et al.*^[54]^ | 2010 | China | Restasis® | BID | 41 | 47.5 (5.9) | 12 months | ①②③④ |
|  |  |  | Artificial tears | BID | 33 | 48.2 (6.3) |  |  |
| Demiryay *et al.*^[55]^ | 2011 | Turkey | Restasis® +Artificial Tears | BID | 22 | 46.6 (12.3) | 4 months | ②③④ |
|  |  |  | Artificial Tears | QID | 20 | 44.3 (14.4) |  |  |
| Prabhasawat *et al.*^[56]^ | 2012 | Thailand | Restasis® | BID | 36 | 48.1 (13.9) | 12 weeks | ①②④ |
|  |  |  | Artificial tears | BID | 34 | 55.0 (13.0) |  |  |
| Kang *et al.*^[29]^ | 2019 | Korea | TJ Cyporin® | BID | 18 | 55.1 (13.5) | 12 weeks | ①②③④ |
|  |  |  | Restasis® | BID | 18 | 53.5 (9.7) |  |  |
| Park *et al.*^[28]^ | 2019 | Korea | TJ Cyporin® | BID | 58 | N/A | 12 weeks | ①②③④ |
|  |  |  | Restasis® | BID | 58 | N/A |  |  |
| Leonardi *et al.*^[31]^ | 2016 | 9 European  countries | Ikervis® | QD | 154 | 60.8 (13.5) | 6 months | ①②③④ |
|  |  |  | Vehicle | QD | 91 | 62.1 (11.8) |  |  |
| Baudouin *et al.*^[32]^ | 2017 | 6 European  countries | Ikervis® | QD | 241 | 57.6 (12.9) | 6 months | ②④ |
|  |  |  | Vehicle | QD | 248 | 58.8 (12.7) |  |  |
| Kim *et al.*^[33]^ | 2017 | Korea | Clacier® | BID | 34 | N/A | 12 weeks | ①②③④ |
|  |  |  | Restasis® | BID | 39 | N/A |  |  |
| Tauber *et al.*^[35]^ | 2018 | United States | Cequa® | BID | 152 | 59.2 (14.6) | 12 weeks | ④ |
|  |  |  | Vehicle | BID | 152 | 59.3 (13.8) |  |  |
| Goldberg *et al.*^[36]^ | 2019 | United States | Cequa® | BID | 371 | 58.4 (14.1) | 12 weeks | ④ |
|  |  |  | Vehicle | BID | 373 | 59.5 (14.7) |  |  |
| Chen *et al.*^[37]^ | 2019 | China | Zirun® | BID | 119 | 46.3 (12.5) | 12 weeks | ①②③④ |
|  |  |  | Vehicle | BID | 115 | 45.0 (12.4) |  |  |
| Wirta *et al.*^[41]^ | 2019 | The United States and Germany | CyclASol® | BID | 51 | 64.3 (10.7) | 16 weeks | ①④ |
|  |  |  | Restasis® | BID | 53 | 62.8 (11.9) |  |  |
|  |  |  | Vehicle | BID | 52 | 61.3 (10.5) |  |  |
| Sheppard *et al.*^[39]^ | 2021 | The United States and Germany | CyclASol® | BID | 162 | 61.5 (13.6) | 12 weeks | ③④ |
|  |  |  | Vehicle | BID | 166 | 61.3 (12.7) |  |  |

Vehicle (the same ophthalmic emulsion formulation without cyclosporine); N/A, data not available; ① Ocular surface disease index (OSDI) score; ② Schirmer’s test (ST) with or without anesthesia; ③ Tear film break-up time (BUT); ④ Treatment-Emergent AEs (TEAEs).

Table 3. League table of results for OSDI and ST score change from baseline.

|  | **ST score change from baseline** | | | | | | |
| --- | --- | --- | --- | --- | --- | --- | --- |
| **OSDI score change from baseline** | **Restasis®** | 1.73 (-3.81,7.27) | 0.45 (-3.53,4.42) | N/A | 0.36 (-4.96,5.68) | 0.48 (-5.07,6.03) | 1.18 (-0.68,3.04) |
|  | 0.51 (-7.07,8.09) | **Zirun**® | 2.18 (-4.64,9.00) | N/A | 1.37 (-6.31,9.05) | 2.21 (-5.18,9.60) | 2.91 (-2.31,8.13) |
|  | -0.32 (-6.45,5.81) | -0.83 (-10.57,8.91) | **TJ** **Cyporin**® | N/A | 0.81 (-5.83,7.45) | -0.03 (-6.86,6.79) | 0.73 (-3.65,5.12) |
|  | -1.42 (-2.96,0.12) | -1.93 (-9.55,5.68) | -1.10 (-7.41,5.21) | **CyclASol®** | N/A | N/A | N/A |
|  | -3.14 (-11.68,5.40) | -3.65 (-15.07,7.76) | -2.82 (-13.33,7.69) | -1.72 (-10.39,6.96) | **Clacier®** | 0.84 (-6.85,8.53) | 1.54 (-4.10,7.18) |
|  | -3.72 (-9.18,1.75) | -4.23 (-13.37,4.91) | -3.40 (-11.60,4.80) | -2.30 (-7.81,3.21) | -0.58 (-10.71,9.56) | **Ikervis®** | 0.70 (-4.53,5.93) |
|  | -4.82 (-6.18,-3.45) | -5.33 (-12.79,2.13) | -4.50 (-10.76,1.77) | **-3.40 (-4.94,-1.86)** | -1.68 (-10.32,6.97) | -1.10 (-6.39,4.19) | **Placebo** |

Each cell contains the odds ratio (OR) and 95% confidence interval for OSDI changes and ST changes; comparisons should be read from left to right. Bold numbers indicate statistically significant differences. OSDI OSDI score change from baseline，STST ST score change from baseline; N/A, data not available.

Table 4 League table of results for BUT score change from baseline and TEAEs.

|  | **BUT change from baseline** | | | | | | | |
| --- | --- | --- | --- | --- | --- | --- | --- | --- |
| **Treatment-emergent AEs** | **Placebo** | -0.02 (-1.04,1.01) | -0.13 (-0.78,0.52) | -0.35 (-1.34,0.64) | -0.44 (-0.92,0.05) | -0.62 (-1.10,-0.14) | -0.74 (-1.84,0.36) | -0.99 (-1.52,-0.46) |
|  | 2.41 (-3.78,8.60) | **Clacier®** | -0.12 (-1.33,1.10) | -0.33 (-1.76,1.09) | **-0.42 (-1.55,0.71)** | **-0.60 (-1.74,0.53)** | **-0.72 (-2.22,0.78)** | **-0.98 (-2.13,0.18)** |
|  | N/A | N/A | **CyclASol®** | **-0.22 (-1.40,0.97)** | **-0.30 (-1.12,0.51)** | **-0.49 (-1.22,0.25)** | **-0.60 (-1.84,0.63)** | **-0.86 (-1.70,-0.02)** |
|  | 2.55 (-4.02,9.12) | 0.14 (-8.89,9.16) | N/A | **Zirun®** | -0.09 (-1.19,1.01) | **-0.27 (-1.37,0.83)** | -0.39 (-1.87,1.09) | **-0.64 (-1.77,0.48)** |
|  | N/A | N/A | N/A | N/A | **Cequa®** | **-0.18 (-0.88,0.52)** | **-0.30 (-1.51,0.92)** | **-0.55 (-1.27,0.16)** |
|  | 1.90 (-0.26,4.06) | 0.51 (-6.04,7.07) | N/A | 0.65 (-5.55,6.85) | N/A | **Restasis®** | **-0.12 (-1.10,0.86)** | **-0.37 (-1.10,0.35)** |
|  | 2.62 (-2.33,7.57) | 0.21 (-7.71,8.13) | N/A | 0.07 (-7.56,7.71) | N/A | 0.72 (-3.73,5.17) | **TJ Cyporin®** | **-0.26 (-1.48,0.97)** |
|  | 0.27 (-4.10,4.63) | 2.14 (-5.43,9.71) | N/A | 2.28 (-5.61,10.16) | N/A | 1.63 (-3.24,6.50) | 2.35 (-4.25,8.95) | **Ikervis®** |

Each cell contains the odds ratio (OR) and 95% confidence interval for BUT changes and TEAEs; the comparison should be read from left to right. Bold numbers indicate statistically significant differences. OSDI BUT score change from baseline, STST ST score change from baseline; N/A, data not available.
